# Interleukin-6 Receptor Blockade can Increase the Risk of Nonalcoholic Fatty Liver Disease: Indications From Mendelian Randomization

**DOI:** 10.3389/fphar.2022.905936

**Published:** 2022-06-07

**Authors:** Shuxuan Li, Lanlan Chen, Guoyue Lv

**Affiliations:** Department of Hepatobiliary and Pancreatic Surgery, First Affiliated Hospital of Jilin University, Changchun, China

**Keywords:** interleukin-6 receptor blockade, interleukin-6 signaling pathway, nonalcoholic fatty liver disease, mendelian randomization, causal inference

## Abstract

**Background:** Interleukin-6 receptor (IL-6R) blockade has been approved for inflammation-associated diseases and whether it is effective in treating non-alcoholic fatty liver disease (NAFLD) is still unknown.

**Methods:** A target-based Mendelian randomization was performed to appraise whether inhibiting the IL-6 signaling pathway *via* IL-6R blockade can reduce the risk of NAFLD. The previously established genetic proxy SNP rs2228145 was mainly used to appraise the therapeutic effects and the genetic-predicted circulating IL-6 level was treated as the exposure with ∼30,000 samples. The genetic association between SNP rs2228145 (A > C) and NAFLD was obtained from non-FinnGen GWAS (1,483 cases and 17,781controls) and FinnGen GWAS (894 cases and 217,898 controls). The causal effects were estimated using a Wald ratio method and were combined using a fixed-effects meta-analysis. Furthermore, the SNP rs12048091 was employed as another proxy in the sensitivity analysis.

**Results:** The positive control analysis suggested the SNP rs2228145 can mimic the effects of IL-6R blockade where inhibiting IL-6 signaling can reduce the risk of rheumatoid arthritis [OR = 0.68 (0.58, 0.80)] and coronary heart disease [OR = 0.75 (0.68, 0.84)]. This Mendelian randomization analysis suggested that IL-6R blockade can adversely increase the risk of NAFLD in the non-FinnGen GWAS [OR = 1.99 (1.27, 3.13)] while not significant in the FinnGen consortium. The fixed-effects meta-analysis indicated inhibiting the IL-6 signaling pathway can reduce the risk of NAFLD [OR = 1.80 (1.26, 2.57)]. When including SNP rs12048091 as the genetic instrument, the meta-analysis using two genetic variants also indicated a similar effect on NAFLD [OR = 1.83 (1.32, 2.53)]. There was no heterogeneity in the whole analysis.

**Conclusion:** Our Mendelian randomization suggested inhibiting the IL-6 signaling pathway *via* IL-6R blockade might increase the risk of NAFLD, suggesting IL-6R should play a protective role in NAFLD.

## Introduction

The prevalence of non-alcoholic fatty liver disease (NAFLD), also termed metabolic associated fatty liver disease (MAFLD), is increasing worldwide and almost 1 billion people are harassed by it (Eslam et al., 2020). Currently, no effective therapeutics have been approved for it though the pan-PPAR agonist lanifibranor displayed the efficacy in phase 2b trial (Francque et al., 2021). Thus, it is of necessity to find therapeutic targets and potential drugs to help ameliorate NAFLD. Interleukin-6 (IL-6) is a pivotal cytokine in inflammation-associated liver disease, and the current evidence suggested it has both pro-inflammation and anti-inflammation effects where IL-6 released from adiposity can promote inflammation and muscle-derived IL-6 can ameliorate inflammation (Giraldez et al., 2021). Furthermore, chronic exposure to high IL-6 levels can increase hepatic gluconeogenesis and result in impaired lipid metabolism (Perry et al., 2015), however, the IL-6 has hepatoprotective effects in acute liver injury (Gao et al., 2020). Also, a recent study suggested IL-6 receptor (IL-6R) can reduce NAFLD-associated inflammation (Skuratovskaia et al., 2021).

It is still unknown whether targeting the IL-6 signaling pathway would have a causal effect on NAFLD through several drugs targeting this pathway that have been approved or displayed clinical potency for metabolic diseases, inflammatory bowel disease (IBD), and liver and gastrointestinal cancer (Giraldez et al., 2021). The rapid developments in Mendelian randomization (MR) have provided strategies to evaluate the causal effect of specific targets with the integration of results derived from genome-wide association studies (GWAS). Several target-based MR investigations have been performed to appraise the therapeutic effects of specific targets and signaling pathways, including the IL-6 signaling pathway (Swerdlow et al., 2012). The target-based MR study used germline genetic variants located in the target gene region as the instruments and it is a quasi-randomized clinical trial (quasi-RCT) since germline genetic variants were usually randomly allocated at conception (Smith and Ebrahim, 2003). Considering the MR estimates often represent a life-long causal effect of exposure on the outcome, this target-based design can evaluate the effect of chronic exposure to a biomarker (Davies et al., 2018).

It should be noted that the inhibiting IL-6 signaling pathway can increase the serum IL-6 level *via* a negative feedback mechanism (Peters et al., 1996). Therefore, we cannot determine whether the hazardous effect of chronic exposure to high IL-6 levels might rise from a loss function of IL-6R. In this target-based MR study, we attempted to explore the causal effect of the IL-6 signaling pathway on NAFLD and furthermore appraise the effect of chronic exposure to high IL-6 levels on NAFLD.

## Methods

### Data Source Description

The genetic associations with circulating IL-6 levels were obtained from the largest GWAS meta-analysis with more than 30,000 European individuals (Folkersen et al., 2020). The IL-6 level was normalized or standardized and this meta-analysis adjusted for population structure and study-specific parameters. The genetic associations with NAFLD were extracted from two independent GWAS summary statistics where one is composed of 1,483 European cases and 17,781 European controls (Anstee et al., 2020), and the other consists of 894 European cases and 217,898 European controls (https://www.finngen.fi/en). The former NAFLD was diagnosed by histopathology and associated laboratory examinations while the latter NAFLD was determined by electronic medical records. In addition, the former was analyzed by a multiple logistic regression by adjusting for the top 5 principal components and the latter was performed under the protocols of FinnGen GWAS round 5 (https://finngen.gitbook.io/documentation/v/r5/). There was no sample overlapping between the data sources of exposure and outcome.

### Selection of Genetic Variants Mimicking IL-6R Blockade

The genetic variants mimicking IL-6R blockade were determined *via* two ways, one is from previous literature and the other was directly from the GWAS summary statistics. Previously, two SNPs located in the gene region coding IL-6R were reported to imitate the effect of inhibiting the IL-6 signaling pathway, namely SNP rs7529229 (Swerdlow et al., 2012) and rs2228145 (Parisinos et al., 2018). The two SNPs are in high linkage disequilibrium among Europeans (LD *R* ^2^ = 0.94). They can increase the proteolysis of IL-6R and result in a reduction in classic signaling, and have a similar effect to tocilizumab, an approved anti-IL6R monoclonal antibody (Swerdlow et al., 2012).

From the GWAS summary statistics of IL-6 level, only three SNP retained based on three criteria (*p*-value < 5 × 10^−8^, minor allele frequency >0.01 and LD R2 < 0.3), including SNP rs2228145, rs12048091 and rs10752641. The SNP rs10752641 was removed further as it is a palindromic one. The LD *R* ^2^ between SNP rs2228145 and rs12048091 was 0.11. To furthermore demonstrate that the selected genetic variants can mimic the effect of inhibiting the IL-6 signaling pathway by IL-6R blockade, we examined their genetic associations with C-reactive protein and fibrinogen, two downstream biomarkers of the IL-6 signaling pathway, in the UK Biobank with 361,194 Europeans. The F statistic was calculated to appraise the weak instrument bias.

### Mendelian Randomization Design and Meta-analysis

**FIGURE 1:**
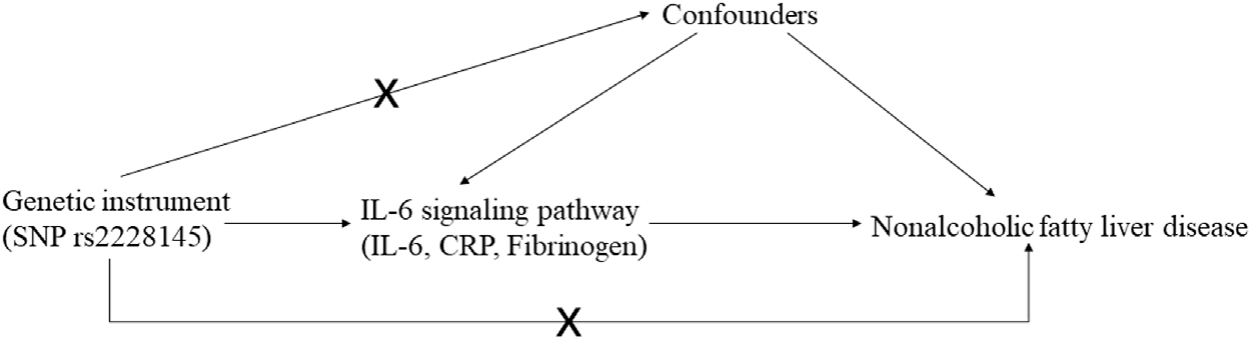
The basic assumptions and main design of this Mendelian randomization.

The MR study should be carried out following three basic criteria: 1) relevance: genetic variants, usually single nucleotide polymorphism (SNP), should be close to the exposure; 2) independence: genetic variants should not be associated with any potential confounders; 3) exclusion-restriction: genetic variants should not be associated with the outcome except *via* the way of exposure (Emdin et al., 2017) (Figure 1). In addition, other assumptions should be satisfied such as linearity and no interaction (Bowden and Holmes, 2019).

Preliminarily, we extracted genetic association with NAFLD from two GWASs for SNP rs2228145 and estimated the effect of IL-6 level on NAFLD using the Wald ratio method. Then, two MR estimates based on the ratio method were combined using a meta-analysis. The fixed-effects model was adopted if there was no heterogeneity. Otherwise, we would use a random-effects model.

### Positive Control Analysis and Sensitivity Analysis

To further guarantee the validity and rigorousness of this MR study, we selected two well-established outcomes as the positive controls such as rheumatoid arthritis (RA) (Okada et al., 2014) and coronary heart disease (CHD) (Nikpay et al., 2015). The causal effect of IL-6R blockade on them was estimated using SNP rs2228145.

As there might be a lack of statistical power with only one SNP, we further included SNP rs12048091 in the sensitivity analysis. The inverse-variance weighted (IVW) method was employed to estimate the causal effect of IL-6 blockade using two SNPs.

### Statistical Analysis and Data Visualization

All statistical analyses and data visualization were performed using the R programming software (R 3.6.0, https://www.r-project.org/). The Wald ratio and IVW methods were provided by the R package “TwoSampleMR” (https://github.com/MRCIEU/TwoSampleMR) (Hemani et al., 2018). The statistical power was assessed by the mRnd (https://cnsgenomics.shinyapps.io/mRnd/) (Brion et al., 2013).

## Results

### Description of Selected Genetic Variants

The SNP rs2228145 (A > C), a missense variant (IL-6R Asp358Ala), was used in the primary analysis, and this variant was associated with increased soluble IL-6R (sIL-6R) and IL-6 levels (Ferreira et al., 2013). In the light of its association with the IL-6 level, its F statistic was 198, much greater than the empirical threshold of 10. The SNP rs12048091 (A > G) was an intron variant located in the IL-6R gene region, however, its biological function is still unknown. Its F statistic was 30, respectively. All genetic associations were aligned to the allele that raises the IL-6 level (Table 1).

**TABLE 1:** Genetic associations with serum interleukin-6 level and F statistics.

SNP	A1	A2	EAF	BETA	SE	P	F
rs2228145	C	A	0.38	0.17	0.012	3.34 × 10^−45^	198.49
rs12048091	A	G	0.82	0.09	0.016	3.95 × 10^−08^	30.01

Note: A1 is the effect allele.

### Genetic Associations With CRP and Fibrinogen

The genetic associations with CRP and fibrinogen confirmed the validity of selected genetic variants (Table 2). As displayed in Table 2, the allele increasing IL-6 level was inversely associated with CRP and fibrinogen for each SNP, suggesting that the SNPs can mimic the inhibition of the classical IL-6 signaling pathway.

**TABLE 2:** Genetic associations with serum CRP and fibrinogen levels and two positive controls (rheumatoid arthritis and coronary heart disease).

SNP	A1	A2	CRP			Fibrinogen			Rheumatoid arthritis			Coronary heart disease		
			BETA	SE	P	BETA	SE	P	BETA	SE	P	BETA	SE	P
rs2228145	C	A	−0.09	0.002	1.00 × 10^−200^	−0.01	0.003	0.001	−0.07	0.015	4.50 × 10^−6^	−0.05	0.010	1.86 × 10^−7^
rs12048091	A	G	−0.06	0.003	4.36 × 10^−65^	−0.01	0.004	0.013	−0.09	0.021	1.30 × 10^−5^	0.02	0.013	0.243

Note: A1 is the effect allele.

When estimating the change of CRP and fibrinogen scaled by the IL-6 level change, we obtained that the CRP level would decrease 0.54 standard deviation (SD) (95% confidence interval (CI): (−0.56, −0.51), *p*-value < 0.001) with per SD increase in IL-6 level and the fibrinogen level would decrease 0.06 [95% CI: (−0.09, −0.02), *p*-value = 0.001) for SNP rs2228145. There were similar results for SNP rs12048091 where the CRP level was reduced 0.62 SD [95% CI: (−0.69, −0.55), *p*-value < 0.001], together with fibrinogen [beta = −0.11 [−0.20, −0.02), *p*-value = 0.013).

### Results of Positive Control Analysis

**FIGURE 2:**
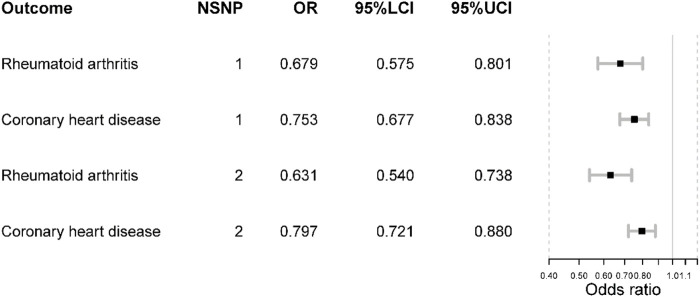
The positive control analysis results. NSNP is the number of SNPs used in analysis and the SNP rs2228145 was used if NSNP = 1. The SNP rs2228145 and rs12048091 were used if NSNP = 2. OR is the odds ratio. 95% LCI is the lower limit of 95% confidence interval while 97%UCI is the upper limit of 95% confidence interval.

The genetic association with positive controls suggested SNP rs2228145 were inversely associated with the risk of RA and CHD. However, the SNP rs12048091 was not associated with CHD. The MR results of the positive control analysis were all statistically significant and concordant with the expectations. Initially, inhibiting IL-6 signaling pathway could reduce the risk of RA [OR = 0.68 (0.58, 0.80), *p*-value < 0.001]. Like RA, the risk of CHD would decrease as well [OR = 0.75 (0.68, 0.84), *p*-value < 0.001]. In the fixed-effects model using two SNPs, we obtained similar results (Figure 2).

### Main Mendelian Randomization Results and Meta-Analysis

**FIGURE 3:**
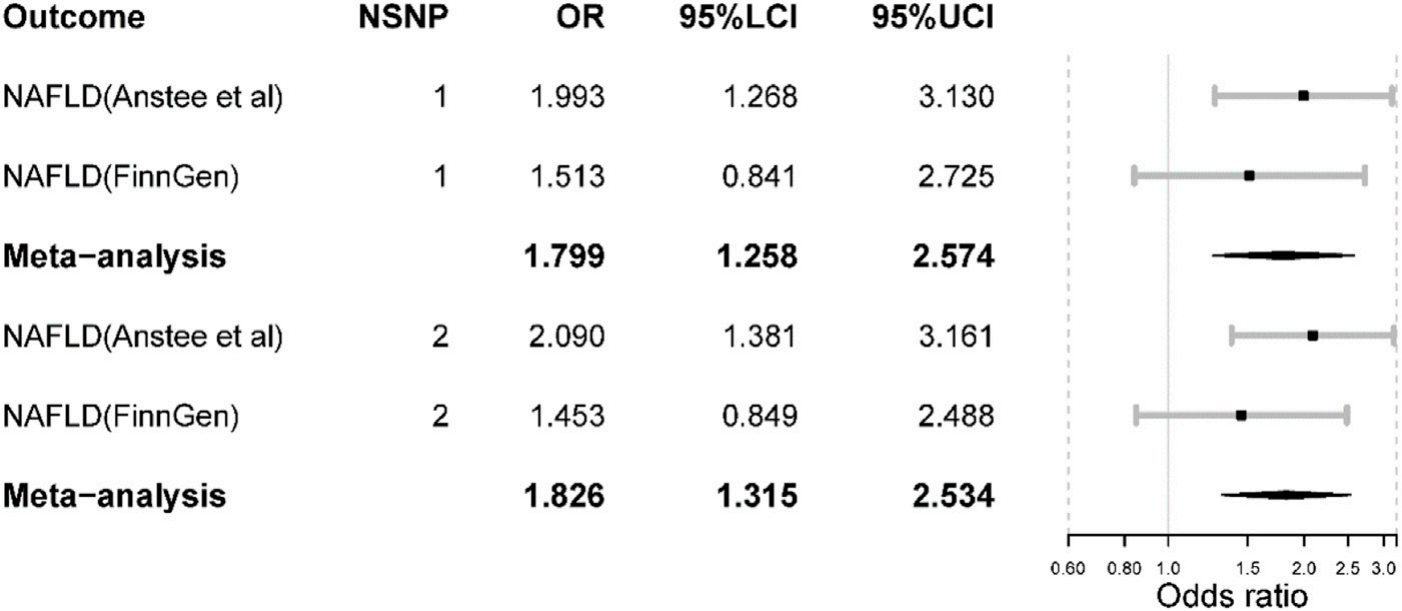
The main Mendelian randomization results. NSNP is the number of SNPs used in analysis and the SNP rs2228145 was used if NSNP = 1. The SNP rs2228145 and rs12048091 were used if NSNP = 2. OR is the odds ratio. 95% LCI is the lower limit of 95% confidence interval while 97%UCI is the upper limit of 95% confidence interval.

In the largest NAFLD GWAS, we observed that the odds of NAFLD would increase if inhibiting IL-6 signaling pathway [OR = 1.99 (1.27, 3.13), *p*-value = 0.003] where the inhibitive effect was measured by IL-6 level and proxied by SNP rs2228145. However, such causal effect was not detected in the FinnGen NAFLD GWAS [OR = 1.51 (0.84, 2.72), *p*-value = 0.167]. In the meta-analysis, the odds of NAFLD would increase [OR = 1.80 (1.26, 2.57), *p*-value = 0.001] (Figure 3). There was no heterogeneity in the meta-analysis (I square = 0; Q *p*-value > 0.05).

When including SNP rs12048091 in the MR analysis, the odds of NAFLD would increase in the largest NAFLD GWAS [OR = 2.09 (1.38, 3.16), *p*-value < 0.001]. Also, the result was not significant in the FinnGen NAFLD GWAS (Figure 3). The meta-analysis yielded a significant result [OR = 1.83 (1.32, 2.53), *p*-value < 0.001]. Compared to the results from the main analysis using only one SNP, the results derived from two SNPs displayed larger effect sizes and statistical power. Furthermore, both MR and meta-analysis results suggested that inhibiting the IL-6 signaling pathway *via* IL-6R blockade could increase the risk of NAFLD.

The statistical powers of the non-FinnGen dataset were 98 and 84% for the FinnGen dataset. In addition, all the other results were above 80%.

## Discussion

This target-based MR analysis gave novel insights into the increased risk of NAFLD when inhibiting the IL-6 signaling pathway *via* IL-6R blockade, especially inhibiting the classic IL-6 signaling pathway.

Currently, there was no direct clinical trial reporting the incidence rate of NAFLD among patients receiving anti-IL6R therapy. Thus, we would concentrate on the effect of anti-IL6R drugs on metabolism, especially on lipid and glucose metabolism. The available evidence indicated that anti-IL6R drugs seemed to be associated with a marginal reduction in glycated hemoglobin (HbA1c) levels (*p* = 0.047) (Lurati et al., 2021) while another trial ruled out this association (Chen et al., 2020). Overall, the anti-IL6R drugs appear not to reduce the risk of diabetes mellitus. The ASCERTAIN trial suggested application of subcutaneous sarilumab and intravenous tocilizumab could increase alanine transaminase (ALT) and lipids, including low-density lipoprotein cholesterol (LDL-C), high-density lipoprotein cholesterol (HDL-C) and triglycerides (TG) (Emery et al., 2019). In addition, other clinical trials also corroborated that tocilizumab treatment could increase the levels of serum lipids, especially LDL-C and HDL-C (Gabay et al., 2016; Hoffman et al., 2019).

However, all these trials correlated the lipid elevation with the increased risk of cardiovascular events and neglected the potential risk of NAFLD. Our study indicated that IL-6R blockade could increase the risk of NAFLD. These trials lent support to our findings since NAFLD is characterized by lipid accumulation and genetically-elevated LDL-C could increase the risk of NAFLD (Liu et al., 2020). Nonetheless, it should be noted that we used CHD as the positive control where IL-6R blockade could reduce the risk of it. Thus, the association between IL-6R blockade, lipid elevation, and NAFLD should be independent of the common IL-6 signaling pathway and might be liver-specific.

The IL-6 signaling can be classified into two categories: 1) classical signaling: IL-6 binds to the signal transducing subunit gp130 in complex with the membrane-bound IL-6R; 2) trans-signaling: IL-6 binds to the gp130 *via* the soluble IL-6R (Schmidt-Arras and Rose-John, 2016). The classical signaling has anti-inflammatory and regenerative properties while the trans-signaling can promote inflammation (Skuratovskaia et al., 2021). Skuratovskaia et al. reported that higher plasma IL-6 level was correlated with a decreased CRP level and IL-6R could reduce chronic inflammation in NAFLD (Skuratovskaia et al., 2021). These findings were consistent with our results as the IL-6 signaling inhibition was scaled by the IL-6 level and this level was inversely associated with CRP and fibrinogen. In this MR study, the SNP rs2228145 promotes the proteolysis of membrane-bound IL-6R and increases the serum level of soluble IL-6R (Ferreira et al., 2013), suggesting this SNP has an inhibitive effect on classical signaling while amplifying the trans-signaling. Thus, it is plausible that we observed an increased risk of NAFLD.

However, the evidence that the impaired lipid metabolism often concurs with high IL-6 levels suggested chronic exposure to high IL-6 levels should promote liver inflammation (Giraldez et al., 2021). In our study, we observed that high IL-6 level caused by IL-6R blockade was associated with the elevated risk of NAFLD. Therefore, we postulated that the chronic exposure to high IL-6 caused by a loss function of IL-6R could promote the chronic inflammation process. A recent study corroborated our findings from another aspect where myeloid-specific IL-6 signaling inhibits liver fibrosis *via* exosomal transfer of antifibrotic miR-223 into hepatocytes, suggesting the protective role of IL-6 signaling in NAFLD (Hou et al., 2021). Although an early study suggested the blockade of interleukin 6 signalings could ameliorate hepatic steatosis *via* modulating insulin resistance, it should be noted that this experiment was performed on mice instead of on human beings and the higher IL-6 level was caused by a high-fat diet (Yamaguchi et al., 2015). Thus, it should be emphasized that the cause of high IL-6 levels should induce different effects and IL-6 signaling effects might vary between mice and people. In addition, the muscle-derived IL-6 could ameliorate inflammation (Pedersen and Febbraio, 2008) while adiposity-derived IL-6 was pro-inflammatory (Coppack, 2001), suggesting the IL-6 effects might vary between tissues. However, the tissue-specificity was not taken into consideration in our study due to data limitations.

Anyhow, this target-based Mendelian randomization suggested the potential risk of NAFLD caused by inhibiting the classic IL-6 signaling, which should be paid attention to in patients receiving anti-IL6R treatments. This study has several strengths as follows: 1) the relatively large sample size of NAFLD; 2) this population-based study added much more robust evidence than animal models or observational studies with small sample sizes. However, several limitations should be pointed out as well. Currently, most of the anti-IL6R monoclonal antibodies usually inhibit both classical signaling and trans-signaling such as tocilizumab (Tanaka et al., 2014). As mentioned above, classical IL-6 signaling is anti-inflammatory while trans-signaling is pro-inflammatory in the liver. Thus, IL-6R monoclonal antibodies should have both anti-/pro-inflammatory effects in the liver and SNP rs2228145 has a single pro-inflammatory effect in the liver, and we cannot equal the MR results to that derived for RCTs. Only two IVs were used in estimating the causal effect, guaranteeing the power and validity of this MR study. In addition, the function of included SNP rs12048091 is unknown and it adds some uncertainty to our results. The causal effects were estimated in European ancestry individuals and the generalizability of our conclusion might not be suitable for other ethnicities.

Last but not least, this target-based MR study assessed the effect of IL-6R blockade proxied by SNP rs2228145 on NAFLD though IL-6R blockade was measured by serum IL-6 level. We cannot obtain the conclusion that the genetically-elevated IL-6 level could increase the risk of NAFLD despite it might be reasonable. Our study implicated that clinical trials focused on anti-IL6R drugs should not neglect the incidence rate of NAFLD. However, clinical trials should be carried out to further corroborate these findings and further investigations should be performed to clarify the tissue-specific effects of IL-6 signaling.

## Conclusion

Our target-based suggested IL-6R blockade might increase the risk of NAFLD and it should be corroborated and paid attention to in further clinical trials.

## Data Availability

Publicly available datasets were analyzed in this study. This data can be found here: https://www.ebi.ac.uk/gwas/.
